# Editorial: Nutritional interventions for tackling micronutrient deficiencies

**DOI:** 10.3389/fnut.2024.1451493

**Published:** 2024-09-19

**Authors:** Aamir Shehzad, Hafiz A. R. Suleria, Sajeela Akram

**Affiliations:** ^1^UniLaSalle, Univ. Artois, ULR7519—Transformations and Agro-ressources, Normandie Université, Mont-Saint-Aignan, France; ^2^School of Agriculture, Food and Ecosystem Sciences, Faculty of Science, The University of Melbourne, Parkville, VIC, Australia; ^3^Department of Human Nutrition and Dietetics, University of Chakwal, Chakwal, Pakistan

**Keywords:** micronutrients, nutritional interventions, food fortification, food supplementation, dietary diversification

Micronutrient deficiencies, often termed “hidden hunger,” represent a significant public health challenge globally, affecting billions of people. These deficiencies, primarily in essential vitamins and minerals like vitamin A, iron, iodine, and zinc, lead to severe health problems, including impaired cognitive development, weakened immunity, and increased morbidity and mortality. Effective nutritional interventions are crucial in mitigating these deficiencies, improving health outcomes, and enhancing quality of life.

One of the most well-documented nutritional interventions is food fortification. This strategy involves adding essential vitamins and minerals to commonly consumed foods. For instance, fortifying wheat flour with iron, folic acid, and B vitamins has been widely implemented with positive outcomes. Studies indicate that iron fortification of staple foods can significantly reduce anemia prevalence, especially in women and children (1). Similarly, salt iodization programs have been instrumental in eliminating iodine deficiency disorders worldwide (2).

Biofortification, another innovative approach, entails breeding crops to naturally increase their nutrient content. Biofortified crops such as zinc rice, iron-rich beans, and vitamin A-enriched sweet potatoes have shown promise in combating specific nutrient deficiencies. Studies have demonstrated that regular consumption of biofortified foods can improve nutritional status and is considered as viable solution for addressing deficiencies and malnutrition (3).

Dietary diversification, promoting the consumption of a variety of nutrient-rich foods, is a sustainable long-term strategy. Encouraging the intake of fruits, vegetables, legumes, nuts, and animal products can address multiple nutrient deficiencies simultaneously. Public health campaigns and educational programs are crucial to changing dietary habits and increasing awareness about the importance of a balanced diet. For instance, the promotion of home gardens has been successful in increasing the availability and consumption of micronutrient-rich foods at the household level (4).

Likewise, food supplementation programs, which involve providing specific micronutrients in pill or liquid form, are particularly effective for addressing acute deficiencies. Vitamin A supplementation has been extensively used to reduce the risk of blindness and mortality in children. According to a Cochrane review, vitamin A supplementation can reduce all-cause mortality by 24% in children aged 6–59 months (5). Similarly, iron supplements are essential for pregnant women to prevent iron deficiency anemia, which can lead to complications such as preterm birth and low birth weight.

Combining all these interventions can lead to more effective outcomes. Integrated programs that include fortification, food supplementation, and dietary diversification, tailored to the specific needs of the population, are essential. For example, a comprehensive program in Guatemala that combined food fortification, supplementation, and education significantly reduced the prevalence of anemia and vitamin A deficiency (6). This Research Topic “*Nutritional interventions for tackling micronutrient deficiencies*” was proposed to gather, evaluate, and publish articles which provide information about various strategies for tackling micronutrient deficiencies and enhance our understanding and knowledge concerning these deficiencies. In this regard, Rehman and Jianglin, related the micronutrient deficiencies with cardiac health through gray rational analyses (GRA) and gray modeling. They underlined that balanced diet comprising principally of fruits and vegetables along with precision nutrition guidelines can reduce cardiac related diseases.

In second research article, Naz et al., targeted food fortification for highlighting the impact of this strategy to tackle anemia through bio-efficacy studies. Anti-anemic effect of iron-fortified jamun leather on biomarkers of anemia and hematology was examined by involving Sprague Dawley rats. The results depicted that these iron-fortified jamun leathers reinstated the serum iron, hemoglobin, ferritin, and hematocrit levels. It was also enumerated that iron fortification in this way had non-toxic effect on rat tissues and organs. It is believed that this study will be beneficial in reducing iron deficiency and addressing anemia, thus improving overall health of individuals. Another research involved the cross-sectional study for assessing dietary intake to determine the role of diet quality and nutrient density on overall health of child and mother during pregnancy (Castro-Barquero et al.). Focus was given on Mediterranean diet to relate it with higher intake of micronutrients during entire period of pregnancy. Experimental results indicated that higher intake of protein, monounsaturated fatty acids, fiber, vitamins, and micronutrients was correlated to higher adherence to Mediterranean diet. Overall, this study is of eminent relevance for women health during pregnancy, particularly the importance of Mediterranean diet for controlling overweight, thus contributing to public health strategy by avoiding nutrient deficiencies.

Aghapour et al., focused on vitamin D deficiency (VDD) in Iran using multiple streams framework (MSF). They performed a policy analysis on vitamin D related policies through qualitative analytical studies employing MSF model. They attributed the prevalence of high hypovitaminosis D to limited intake of foods rich in vitamin D and people's insufficient exposure to sunlight during the day which was associated with non-communicable diseases (NCDs) resulting in increased economic costs. It was concluded that proper implementation of VDD policies by high-level policymakers can facilitate to combat vitamin D deficiency.

However, successful implementation of various interventions requires addressing underlying issues such as poverty, food insecurity, and lack of access to healthcare. Policies aimed at improving food systems, ensuring equitable distribution of resources, and enhancing healthcare infrastructure are vital. Moreover, monitoring and evaluation mechanisms are necessary to assess the impact of interventions and make data-driven adjustments.

In conclusion, nutritional interventions play a critical role in addressing micronutrient deficiencies. Fortification, biofortification, dietary diversification and food supplementation supported by robust policies and public health initiatives, can significantly improve micronutrient status and overall health outcomes. Continued research, policy support, and community engagement are essential to sustain and expand these efforts, ultimately achieving better health for all.

## Author contributions

AS: Conceptualization, Writing – original draft, Writing – review & editing. HS: Writing – original draft, Writing – review & editing. SA: Writing – review & editing.
